# Complications and Comorbidities in COVID-19 Patients: A Comparative study

**DOI:** 10.7759/cureus.28614

**Published:** 2022-08-31

**Authors:** Omair H Al-Hussain

**Affiliations:** 1 Otolaryngology - Head and Neck Surgery, Imam Mohammad Ibn Saud Islamic University, Riyadh, SAU

**Keywords:** hypertension, diabetes, complications, co-morbidities, symptoms, covid-19

## Abstract

Background

COVID-19 has rapidly spread across the world at an unprecedented rate. The outbreak of COVID-19 infection has led to a significant health burden on infected patients, especially those with existing comorbidities. The aim of this study is to find the most prevalent symptoms, comorbidities, and complications developed during the course of the disease and outline the most prevalent symptoms among the baseline and clinical characteristics of the patients.

Methods

This is a retrospective study that was performed on the data obtained from medical records of 3999 COVID-19 patients from Prince Mohammed Bin Abdulaziz Hospital in Riyadh, Saudi Arabia. Demographic data, clinical symptoms, and comorbidities were noted on the day of hospital admission. Complications developed during the COVID -19 infection were also observed.

Results

The average age was 49.55 ± 14.75 years and 73.77% of the study population were male patients. The average Body Mass Index (BMI) of the patients was 29.48 ± 6.94. Fever and cough were the most common symptoms (85.85%) followed by shortness of breath (83.25%). Other reported symptoms were diarrhoea (17.43%), fatigue (16.2%), vomiting (15.38%), headache (15.23%), sore throat (9.3%), and nausea (8.5%) The most common comorbidity recorded was diabetes mellitus (DM) (39.51%), followed by hypertension (HTN) (33.91%), and asthma (9.45%). In COVID-19 patients with comorbidities, 61.90% developed complications of pneumonia, 8.73% had Acute Respiratory Distress Syndrome (ARDS), 7.25% developed pneumonia and ARDS concurrently, while 0.4% of the total patients had septic shock.

Conclusion

The symptoms of fever, cough, and shortness of breath were higher in individuals with hypertension and diabetes mellitus, and more prevalent in complications of pneumonia, acute respiratory illness, and septic shock.

## Introduction

A novel coronavirus caused viral pneumonia in a cluster of patients in December 2019. The International Committee on Taxonomy of Viruses officially named the novel coronavirus Severe Acute Respiratory Syndrome Coronavirus-2 (SARS-CoV-2) whereas the disease caused by the SARS-CoV-2 was named Coronavirus Disease 2019 (COVID-19) by the World Health Organization [1, 2]. Since the emergence of COVID-19 as a pandemic, various studies have been conducted on the disease. However, there are still major limitations in our knowledge and understanding of the disease with respect to demographic characteristics, associated comorbidities, and ensuing complications [3].

There is increasing evidence on the gender disparity of COVID-19 that reports an increased occurrence among males more than females. According to a study in China, out of 44,672 COVID-19 cases extracted from the Infectious Disease Information System, 51.4% of the patients were male [4]. Besides an increased incidence of the disease among males as well as an increase in the severity with unfavorable clinical outcomes were common among males [5].

The clinical presentation of COVID-19 infection may occur with the absence of any signs and symptoms and is thus considered asymptomatic. However, it can also manifest with a wide array of clinical characteristics. Although COVID-19 can display a wide range of symptoms, fever, cough, and shortness of breath or dyspnea occur most frequently due to poor blood oxygenation [6]. Moreover, other clinical characteristics can be present. Gastrointestinal symptoms include loss of appetite, nausea, vomiting, diarrhea, and loss of taste. Symptoms of the upper respiratory tract can include rhinorrhea, loss of smell, and sore throat. In addition, subjective constitutional symptoms such as fatigue and malaise, myalgia have also been reported among infected patients [6, 7].

Comorbidities are considered risk factors for severe outcomes of COVID-19. COVID-19 patients with chronic comorbid conditions suffer from a more severe form of the disease and experience worse progression and clinical outcomes as compared to patients without comorbid conditions [3, 8]. The literature has reported various chronic comorbidities such as diabetes mellitus (DM), hypertension (HTN), and asthma [3]. However, the comorbidities that can lead to severe respiratory complications in COVID-19 still warrant further research [8]. The aim of this study is to explore COVID-19 patients and their associated complications.

## Materials and methods

This is a retrospective chart review study that was conducted on patients admitted with COVID-19 infection in Saudi Arabia between March 2020 to April 2021 in Prince Mohammed Bin Abdulaziz Hospital in Riyadh, Saudi Arabia. The admission criteria were based on the institutional guidelines of the severity. The data set constitutes 3999 COVID-19 patients. Demographic information, clinical symptoms, comorbidities including DM, HTN, asthma, and chronic rhinosinusitis (CRS), and clinical outcomes of patients were studied. The study included all the confirmed cases of COVID -19 patients. The aim of this study was to find the most prevalent symptoms occurring in COVID-19, comorbidities, and complications developed during the infection and analyze the most prevalent symptoms among the baseline and clinical characteristics of patients. The study was approved by the Institutional Review Board (IRB) of Prince Mohammed Bin Abdulaziz Hospital in Riyadh and the research was performed in line with the ethical standards of the Declaration of Helsinki.

The information was collected from the hospital medical records and included demographic information, clinical symptoms and signs, and complications that developed during the COVID-19 disease. The data was entered into Microsoft Excel (Microsoft Corporation, Redmond, USA). Variables were then imported into Statistical Package for Social Sciences (SPSS) version 26 (IBM Corp., Armonk, USA) for statistical analysis. Qualitative variables were expressed as numbers and percentages, while quantitative variables were expressed as means, medians, and Interquartile Ranges (IQR).

## Results

As shown in Table 1, the number of male patients was higher than female patients The mean age for male patients was 48.83 ± 14.16 years and the median was 53 years, while the median age for female patients was 51.59 years. It has been observed that 154 (3.85%) patients had no symptoms, while most patients 3845 (96.15%) had symptomatic COVID-19. Fever and cough were the most common symptoms reported (85.85%), followed by shortness of breath (83.25%). Diarrhea (17.43%), fatigue (16.2%), vomiting (15.38%), headache (15.23%), sore throat (9.3%), and nausea (8.5%) were among other commonly reported symptoms. Anorexia (8 %), chest pain (7.28 %), malaise (6.35 %), abdominal pain (4.78 %), anosmia (2.5 %), loss of taste (2.4 %), dizziness (2 %), runny nose (1.08 %), rhinorrhea (0.28 %), and palpitations (0.28 %) were also reported. Symptoms such as cough (86.5%), shortness of breath (84%) and fever (80.7%), were higher in male patients as compared to female patients wihtout any statsitical significance found regarding gender (Table 2). 

**Table 1:** Baseline characteristics of patients admitted with COVID-19 IQR: Inter Quartile Range, SD: Standard Deviation, BMI: Body Mass Index, n: Numbers

	Frequency (n)	Percentage
Gender		
Female	1049	26.23
Male	2950	73.77
Age		
MEAN ± SD	49.55 ± 14.75	
Median (IQR)	49 (22)	
BMI		
MEAN ± SD	29.48 ± 6.94	
Median (IQR)	28 (8)	
Asymptomatic	154	3.85
Symptomatic	3845	96.15

**Table 2:** Clinical characteristics of patients admitted with COVID-19

Symptoms	Frequency	Percentage
Fever	3433	85.85%
Cough	3433	85.85%
Shortness of breath	3329	83.25%
Diarrhea	697	17.43%
Fatigue	648	16.2%
Vomiting	615	15.38%
Headache	609	15.23%
Sore throat	372	9.3%
Nausea	340	8.5%
Loss of appetite	320	8%
Malaise	254	6.35%
Chest pain	291	7.28%
Abdominal pain	191	4.78%
Loss of smell	100	2.5%
Loss of taste	96	2.4%
Dizziness	80	2%
Runny nose	43	1.08%
Rhinorrhoea	11	0.28%
Palpitation	11	0.28%

Regarding comorbidities, the most common comorbidity was DM (39.51%), followed by HTN (33.91%) and asthma (9.45%), while CRS was the least common comorbidity (0.50%) (Table 3). Fever and cough were found to be higher in patients who had DM (88.1%, 88%) and HTN (87.2%). Shortness of breath was detected in patients who had DM (89.9%), asthma (88.4%) and HTN (88%). Pneumonia was higher in patients who had comorbidities such as CRS (78.9%), DM (70.7%), HTN (69.9%), and asthma (63.2%). Acute Respiratory Distress Syndrome (ARDS) was more common in patients who had HTN (12.2%), DM (11.4%). asthma (10.8%). Septic shock was higher in COVID-19 patients with HTN and DM (0.8%) (Table 4). There was no statistical significance found between comorbidities and complications. Regarding diabetes, 340 (21.5%) patients required ICU admission, while DM mortality rate accounted for 387 (24.5%). Meanwhile, hypertensive patients showed higher mortality rate (461, 33.9%), with an ICU admission rate of 284 (18.3%). Lastly, only 120 (3%) patients with DM and HTN were admitted to the ICU, reporting a high mortality rate of 68 (56.6%).

**Table 3:** Prevalence of comorbidities of patients admitted with COVID-19 n: numbers

Comorbidities	Frequency (n)	Percentage (%)
Diabetes Mellitus	1580	39.51
Hypertension	1356	33.91
Asthma	378	9.45
Chronic Rhinosinusitis	20	0.50

**Table 4:** Demographic and clinical characteristics of COVID-19 patients stratified by different comorbidities n: numbers, SOB: shortness of breath, ARDS: acute respiratory distress syndrome

	Diabetes Mellitus		Hypertension	
	Yes n (%)	No n (%)	Yes n (%)	No n (%)
Sex				
Male	1137 (71.8%)	1813 (75.1)	910 (67.1%)	2040 (77.2%)
Female	447 (28.2%)	602 (24.9%)	446 (32.9%)	603 (22.8%)
Smoker	33 (2.1%)	78 (3.2%)	33 (2.4%)	78 (3%)
Asymptomatic	30 (1.9%)	124 (5.1%)	35 (2.6%)	119 (4.5%)
Symptomatic	1554 (98.1%)	2291 (94.9%)	1321 (97.4%)	2524 (95.5%)
Symptoms				
Fever	1396 (88.1%)	2037 (84.3%)	1183 (87.2%)	2250 (85.1%)
Cough	1394 (88%)	2039 (84.4%)	1188 (87.6%)	2245 (84.9%)
SOB	1424 (89.9%)	1905 (78.9%)	1193 (88%)	2136 (80.8%)
Diarrhea	227 (14.3%)	470 (19.5%)	210 (15.5%)	487 (18.4%)
Fatigue	233 (14.7%)	415 (17.2%)	202 (14.9%)	446 (16.9%)
Vomiting	236 (14.9%)	379 (15.7%)	190 (14%)	425 (16.1%)
Headache	198 (12.5%)	411 (17%)	183 (13.5%)	426 (16.1%)
Sore throat	108 (6.8%)	264 (10.9%)	102 (7.5%)	270 (10.2%)
Nausea	123 (7.8%)	217 (9%)	96 (7.1%)	244 (9.2%)
Loss of appetite	142 (9%)	178 (7.4%)	112 (8.3%)	208 (7.9%)
Malaise	77 (4.9%)	177 (7.3%)	72 (5.3%)	182 (6.9%)
Chest pain	108 (6.8%)	183 (7.6%)	94 (6.9%)	197 (7.5%)
Abdominal pain	65 (4.1%)	126 (5.2%)	52 (3.8%)	139 (5.3%)
Loss of smell	19 (1.2%)	81 (3.4%)	23 (1.7%)	77 (2.9%)
Loss of taste	19 (1.2%)	77 (3.2%)	23 (1.7%)	73 (2.8%)
Dizziness	22 (1.4%)	58 (2.4%)	23 (1.7%)	57 (2.2%)
Runny nose	7 (0.4%)	36 (3.5%)	6 (0.4%)	37 (1.4%)
Rhinorrea	3 (0.2%)	8 (0.3%)	2 (0.1%)	9 (0.3%)
Palpitation	4 (0.3%)	7 (0.3%)	4 (0.3%)	7(0.3%)
Developed conditions				
Pneumonia	1120 (70.7%)	1327 (54.9%)	948 (69.9%)	1499 (56.7%)
ARDS	181 (11.4%)	168 (7%)	166 (12.2%)	183 (6.9%)
Septic shock	12 (0.8%)	5 (0.2%)	11 (0.8%)	6 (0.2%)

## Discussion

In our study, 3999 patients were confirmed to be infected with COVID-19. We analyzed the baseline characteristics, clinical symptoms, and complications developed during the COVID-19 of 3999 patients admitted to Prince Mohammed Bin Abdulaziz Hospital in Riyadh, Saudi Arabia. According to our results, the distribution of COVID-19 among genders shows a higher predisposition in males with 73.77% as compared to females which were only 26.23% without any statistical difference. The median age of the male patients was higher than that of female patients. Similarly, a retrospective study by Kushwaha et al. reported 112,860, COVID-19 patients, in which males were more commonly infected than females (65.39% vs 34.61%). The median age of males with COVID-19 was 39.47±17.59 years, whereas the median age of females was 36.85±18.5 years [9, 10]. The most prevalent clinical symptoms were fever and cough (85.5%), followed by shortness of breath (83.25%). This goes in line with a multicentre, retrospective cross-sectional study by Alahmari et al. which was conducted in Saudi Arabia. In their study of 240,474 patients, COVID-19 occurred predominantly in male patients (71.7%). Furthermore, the presence of symptoms was reported in 63% of cases, in which the most common symptoms were fever in 85.2% and cough in 85% of the study group [11]. Likewise, a retrospective study in China on 262 patients reported the most common symptom of COVID-19 was shortness of breath. The most frequently occurring symptoms were fever in 82.1% of patients and cough in 45.8% of patients, whereas dyspnea occurred in only 6.9% of patients [12]. Another study on 370,000 confirmed symptomatic COVID-19 cases was reported by the Centers for Disease Control and Prevention in the United States. Patients in the study reported a wide spectrum of symptoms similar to our study. Nausea and anorexia were reported in 12% of patients, and anosmia, dysgeusia, abdominal pain, and rhinorrhoea each are reported in less than 10% of patients [13].

Huang et al. described the clinical characteristics of 41 confirmed cases of COVID-19 patients, of which 13 (32%) of them had underlying conditions such as cardiovascular disease, diabetes, hypertension, and chronic obstructive pulmonary disease [14]. Similarly, Wang et al. published findings from 138 COVID-19 cases, indicating that 64 (46.4 %) of them had comorbidities [15]. According to our findings, the most common comorbidity among patients was DM (39.51%), followed by HTN (33.91%) and asthma (9.45%), while CRS was the least common comorbidity (0.50%). A systematic review conducted by Baradaran et al. found that the most common comorbidity among confirmed COVID-19 patients was HTN, which was detected in the fifth of the cases, while DM affected nearly 10% of the patients. On the other hand, other comorbidities accounted for less than 10% of the total [16]. Similarly, a systematic review and meta-analysis by Singhal et al. reported that HTN was the most common comorbidity (48, 95% Confidence Interval (CI) 36-60%) followed by DM (22, 95% CI 13-32%) [17]. Furthermore, the severity of COVID-19 was found to be higher in patients with diabetes [18, 19]. A large meta-analysis conducted by the Brazilian Diabetes Society Study Group discovered that DM and HTN were the most important risk factors for the severity and mortality among COVID-19 patients [20]. As Saudi Arabia is among the top ten countries with the highest prevalence of DM worldwide [21], it is crucial to look at the differences between diabetic and non-diabetic COVID-19 patients. Meanwhile, the average BMI of the patients was higher than the normal rate, which may be a contributing factor to the increased rate of DM. In our study, the prevalence of DM was higher in COVID-19 and patients experienced a higher rate of complications such as pneumonia, ARDS, and septic shock compared to the patients without DM. These findings largely support previous observations from several studies associating DM with the severity of COVID-19 and clinical outcomes [22, 23]. From the related studies and current study findings, it has been illustrated those underlying diseases such as diabetes can increase infectivity and develop serious complications. A comparative study conducted in the United Arab Emirates among COVID-19 patients with and without DM showed a significant increase in the severity that led to critical complications and outcomes [24].

Patients with severe COVID -19 have been found to have significantly higher comorbidities, such as HTN [25]. The risk of getting a severe form of COVID-19 is significantly increased in people with HTN [26, 27]. According to a report on COVID-19 patients in Italy, the median age of COVID-19 patients who died in Italy was 81 years, and 73% had HTN [28]. Our study also reveals that 33.91% of the patients had HTN, and the major clinical manifestations, such as fever (87.2%), cough (87.6%), and shortness of breath (88%), were found to be higher in patients with HTN compared to non-hypertensive patients. In our study, 69.9% of HTN patients developed pneumonia followed by ARDS (12.2 %) and septic shock (0.8%).

It is speculated that patients with asthma are more likely to get infected with COVID-19 and develop more serious complications. However, according to our findings, only 9.45% of COVID-19 patients had pre-existing asthma. There is currently no convincing evidence correlating the increased rate of COVID-19 infection in asthmatic patients. However, other behavioral factors, such as smoking and associated comorbidities, may impact the risk of COVID-19 and its complications [29-31]. Thus, COVID-19 patients with known comorbidities of DM and HTN can have a poor outcome from COVID-19, leading to a poor prognosis [20].

A few limitations that were associated with our research was the type of the study, as it was descriptive and non-randomized. As a result, clinical results were not as precise as those seen in prospective trials. As this was a single-center study, the results cannot be externally validated and are not representative of the general population. Secondly, COVID-19's severity was not graded. As a result, the percentage of patients in each grade could not be calculated. Furthermore, other variables were not explored, such as intubation rate, length of intubation, concurrent infections, and history of smoking.

## Conclusions

Gender disparity is found in COVID-19 as males are more commonly affected than women. The predominant clinical symptoms are fever, cough, and shortness of breath, but gastrointestinal symptoms of diarrhea, vomiting and constitutional symptoms of fatigue and headache are also very common. There is conflicting evidence on the most common chronic comorbidity in COVID-19, as the majority of the studies report diabetes mellitus as the most common comorbidity, however, a few studies report hypertension to be more common. Nevertheless, COVID-19 patients with DM are at a higher risk for more severe forms of COVID-19 and are also highly susceptible to complications of COVID-19 such as pneumonia and ARDS.

## References

[1] Organization WH (2020). Naming the coronavirus disease (COVID-19) and the virus that causes it. Brazilian Journal of Implantology and Health Sciences.

[2] Shereen MA, Khan S, Kazmi A, Bashir N, Siddique R (2020). COVID-19 infection: origin, transmission, and characteristics of human coronaviruses. J Adv Res.

[3] Sanyaolu A, Okorie C, Marinkovic A (2020). Comorbidity and its impact on patients with COVID-19. SN Compr Clin Med.

[4] Mukherjee S, Pahan K (2021). Is COVID-19 gender-sensitive?. J Neuroimmune Pharmacol.

[5] Acheampong DO, Barffour IK, Boye A, Aninagyei E, Ocansey S, Morna MT (2020). Male predisposition to severe COVID-19: review of evidence and potential therapeutic prospects. Biomed Pharmacother.

[6] Shirani K, Sheikhbahaei E, Torkpour Z (2020). A narrative review of COVID-19: the new pandemic disease. Iran J Med Sci.

[7] Larsen JR, Martin MR, Martin JD, Kuhn P, Hicks JB (2020). Modeling the onset of symptoms of COVID-19. Front Public Health.

[8] Ng WH, Tipih T, Makoah NA (2021). Comorbidities in SARS-CoV-2 patients: a systematic review and meta-analysis. mBio.

[9] Li LQ, Huang T, Wang YQ (2020). COVID-19 patients' clinical characteristics, discharge rate, and fatality rate of meta-analysis. J Med Virol.

[10] Kushwaha S, Khanna P, Rajagopal V, Kiran T (2021). Biological attributes of age and gender variations in Indian COVID-19 cases: A retrospective data analysis. Clin Epidemiol Glob Health.

[11] Alahmari AA, Khan AA, Elganainy A, Almohammadi EL, Hakawi AM, Assiri AM, Jokhdar HA (2021). Epidemiological and clinical features of COVID-19 patients in Saudi Arabia. J Infect Public Health.

[12] Tian S, Hu N, Lou J (2020). Characteristics of COVID-19 infection in Beijing. J Infect.

[13] McIntosh K (2022). COVID-19: clinical features. UpToDate [Internet].

[14] Huang C, Wang Y, Li X (2020). Clinical features of patients infected with 2019 novel coronavirus in Wuhan, China. Lancet.

[15] Wang D, Hu B, Hu C (2020). Clinical characteristics of 138 hospitalized patients with 2019 novel coronavirus-infected pneumonia in Wuhan, China. JAMA.

[16] Baradaran A, Ebrahimzadeh MH, Baradaran A, Kachooei AR (2020). Prevalence of comorbidities in COVID-19 patients: a systematic review and meta-analysis. Arch Bone Jt Surg.

[17] Singhal S, Kumar P, Singh S, Saha S, Dey AB (2021). Clinical features and outcomes of COVID-19 in older adults: a systematic review and meta-analysis. BMC Geriatr.

[18] Guo W, Li M, Dong Y (2020). Diabetes is a risk factor for the progression and prognosis of COVID-19. Diabetes Metab Res Rev.

[19] Zhou F, Yu T, Du R (2020). Clinical course and risk factors for mortality of adult inpatients with COVID-19 in Wuhan, China: a retrospective cohort study. Lancet.

[20] de Almeida-Pititto B, Dualib PM, Zajdenverg L, Dantas JR, de Souza FD, Rodacki M, Bertoluci MC (2020). Severity and mortality of COVID 19 in patients with diabetes, hypertension and cardiovascular disease: a meta-analysis. Diabetol Metab Syndr.

[21] Aguiree F, Brown A, Cho NH (2013). IDF Diabetes Atlas, 6th Ed. https://dro.deakin.edu.au/view/DU:30060687.

[22] Varikasuvu SR, Dutt N, Thangappazham B, Varshney S (2021). Diabetes and COVID-19: a pooled analysis related to disease severity and mortality. Prim Care Diabetes.

[23] Miller LE, Bhattacharyya R, Miller AL (2020). Diabetes mellitus increases the risk of hospital mortality in patients with Covid-19: systematic review with meta-analysis. Medicine (Baltimore).

[24] Elemam NM, Hannawi H, Salmi IA, Naeem KB, Alokaily F, Hannawi S (2021). Diabetes mellitus as a comorbidity in COVID-19 infection in the United Arab Emirates. Saudi Med J.

[25] Zhang P, Zhu L, Cai J (2020). Association of inpatient use of angiotensin-converting enzyme inhibitors and angiotensin II receptor blockers with mortality among patients with hypertension hospitalized with COVID-19. Circ Res.

[26] Fang X, Li S, Yu H (2020). Epidemiological, comorbidity factors with severity and prognosis of COVID-19: a systematic review and meta-analysis. Aging (Albany NY).

[27] Bai Y, Yao L, Wei T, Tian F, Jin DY, Chen L, Wang M (2020). Presumed asymptomatic carrier transmission of COVID-19. JAMA.

[28] Remuzzi A, Remuzzi G (2020). COVID-19 and Italy: what next?. Lancet.

[29] Lombardi C, Gani F, Berti A, Comberiati P, Peroni D, Cottini M (2021). Asthma and COVID-19: a dangerous liaison?. Asthma Res Pract.

[30] Novak N, Cabanillas B (2020). Viruses and asthma: the role of common respiratory viruses in asthma and its potential meaning for SARS-CoV-2. Immunology.

[31] Caminati M, Lombardi C, Micheletto C (2020). Asthmatic patients in COVID-19 outbreak: few cases despite many cases. J Allergy Clin Immunol.

